# Supplementary figures and images for: Genotype by environment interaction, correlation, AMMI, GGE biplot and cluster analysis for grain yield and other agronomic traits in sorghum (Sorghum bicolor L. Moench)
Source: PLoS One. 2021 Oct 5;16(10):e0258211. doi: 10.1371/journal.pone.0258211 (PMC8491923; doi:10.1371/journal.pone.0258211)

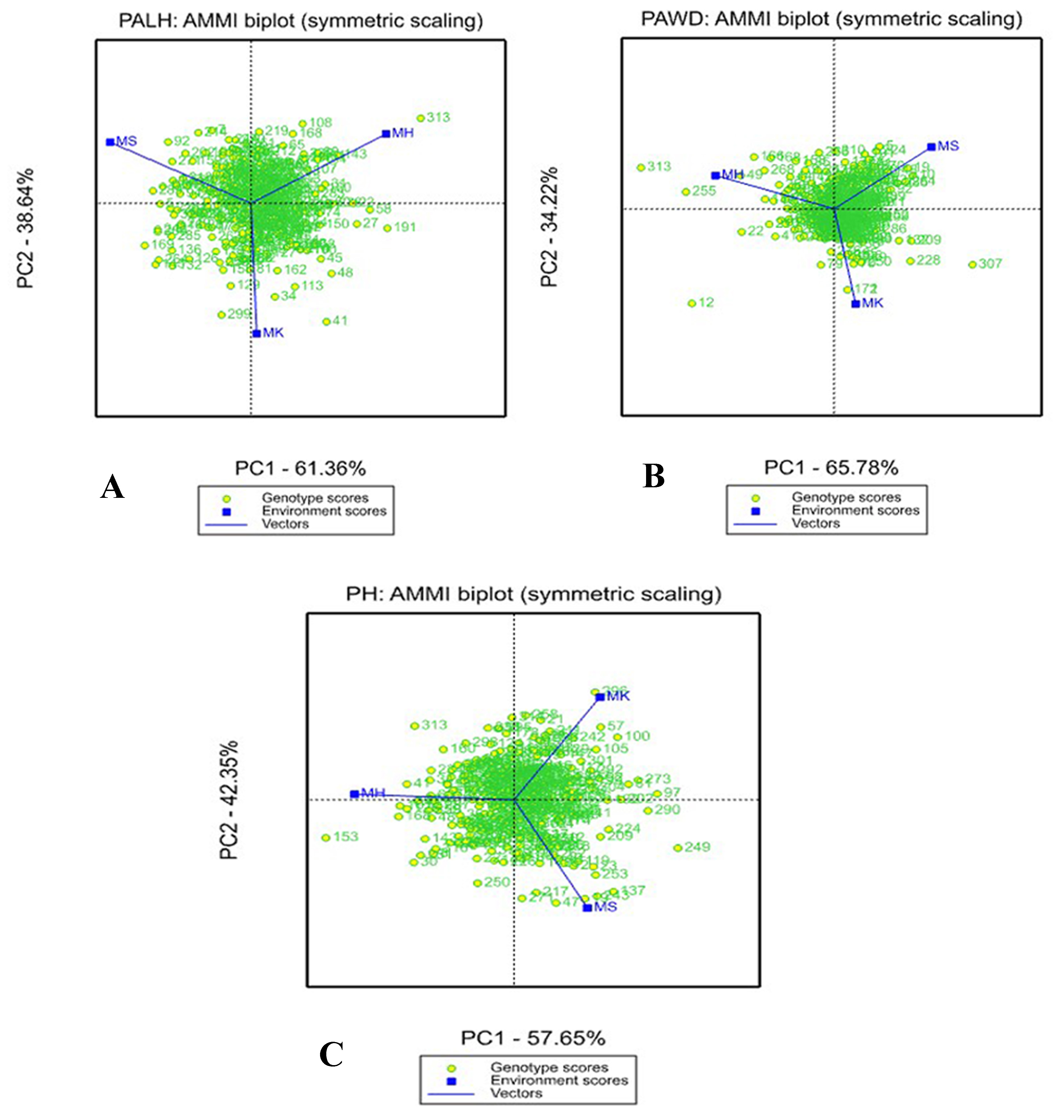

Supplement: S1 Fig — Genotypes placed close to a given environment, had top performance in that environment. Each vector shows the discrimination power of the environment (the longer the vector the more discrimination power that environment has). (TIF) [file pone.0258211.s001.tif]

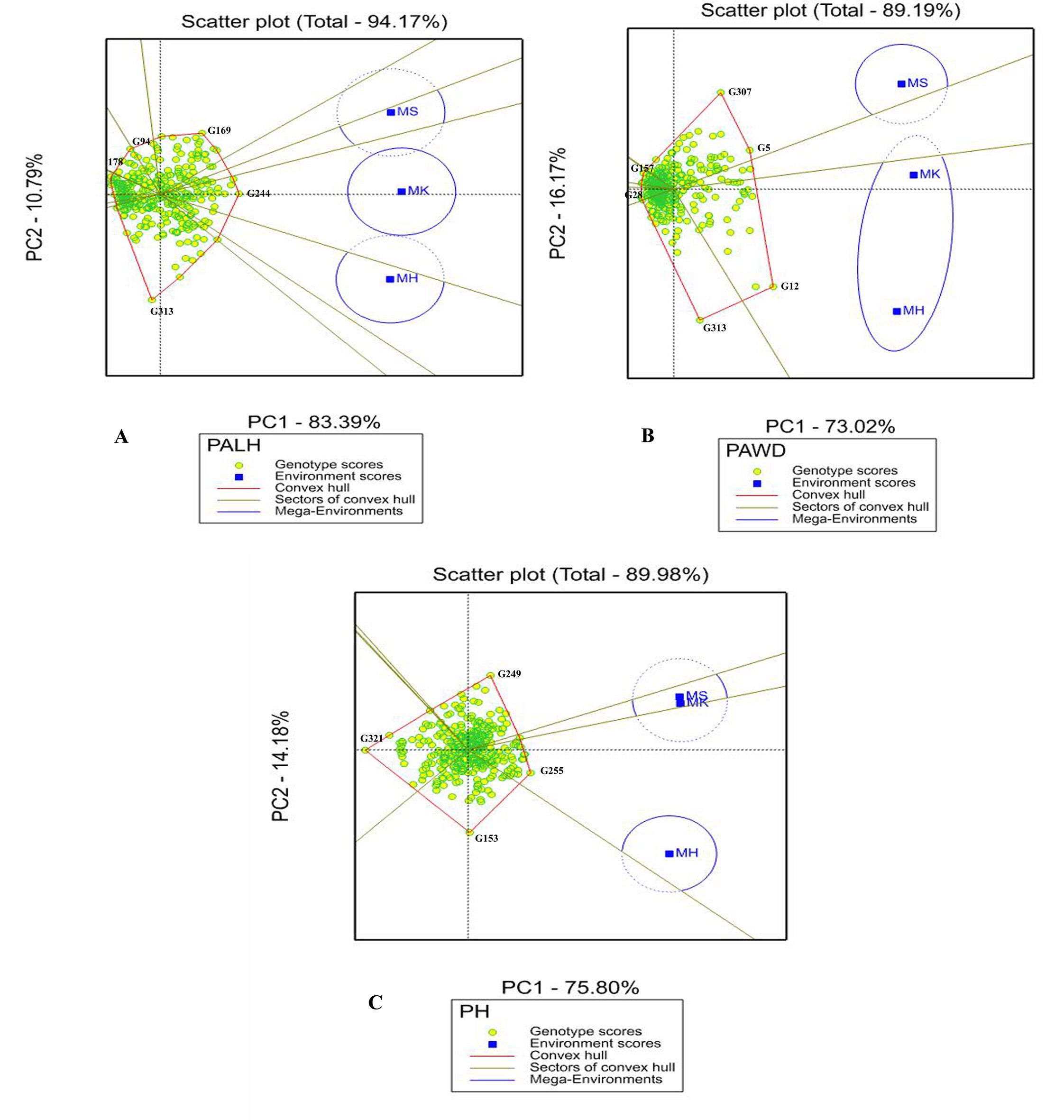

Supplement: S2 Fig — The vertex genotypes on convex hull (Polygon) are the best in each mega environment for the corresponding trait. (TIF) [file pone.0258211.s002.tif]

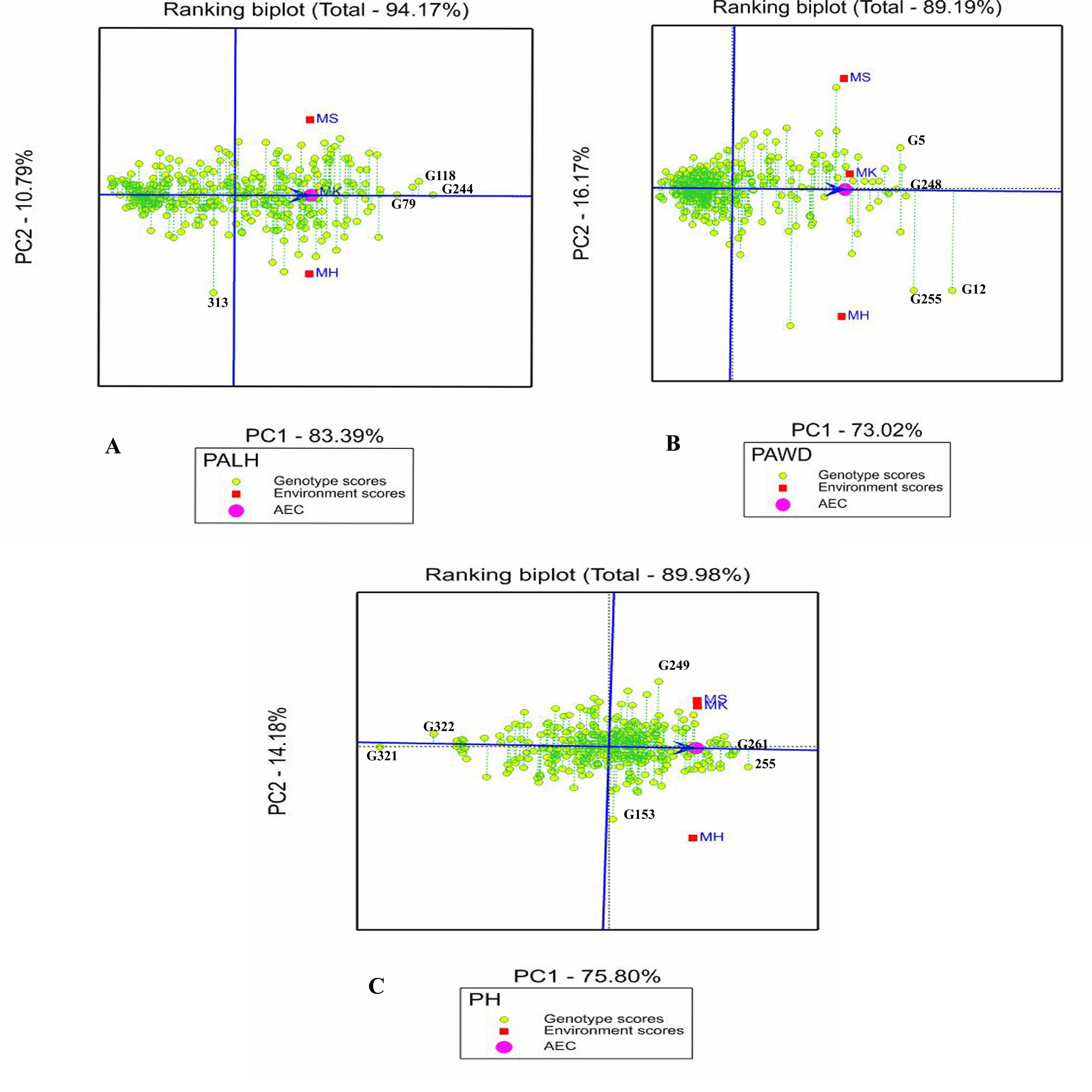

Supplement: S3 Fig — The blue arrowhead line that passes through the origin shows higher mean performance of a genotype and the green dotted lines extending from the blue arrowhead line show the stability of the genotypes (the shorter the dotted line the higher the stability of the genotype). (TIF) [file pone.0258211.s003.tif]

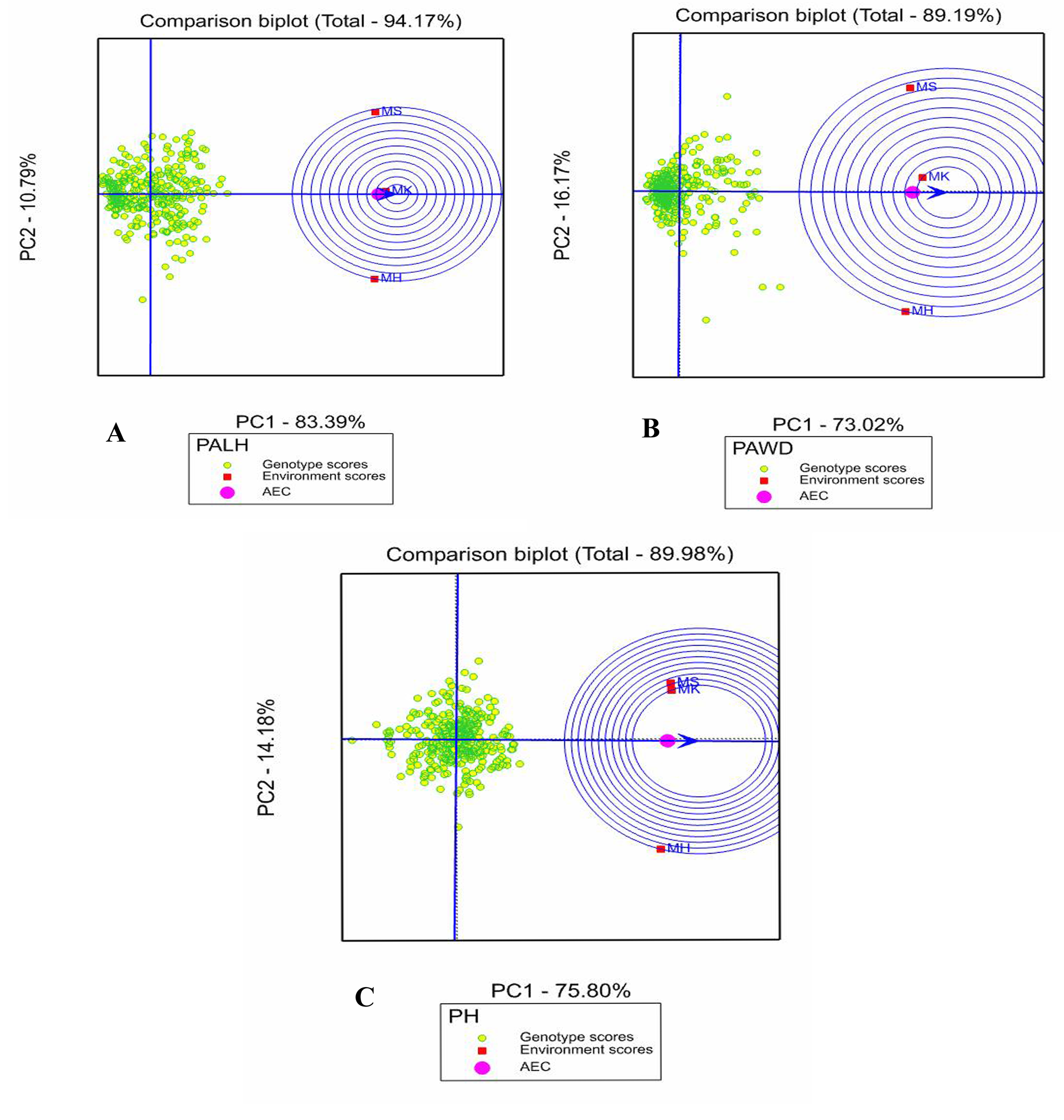

Supplement: S4 Fig — The concentric circles on the biplot show the distance of the environments from AEC and the biplot origin. The ideal environment is the one that is close to the center of the concentric circles. (TIF) [file pone.0258211.s004.tif]
