# Supplementary material for: Genotype by environment interaction, correlation, AMMI, GGE biplot and cluster analysis for grain yield and other agronomic traits in sorghum (Sorghum bicolor L. Moench)
Source: PLoS One. 2021 Oct 5;16(10):e0258211. doi: 10.1371/journal.pone.0258211 (PMC8491923; doi:10.1371/journal.pone.0258211)
Supplement: Attachment — Submitted filename: Response to Reviewers.docx [file pone.0258211.s007.docx]

**A rebuttal letter**

Journal Requirements:

When submitting your revision, we need you to address these additional requirements.

1. Please ensure that your manuscript meets PLOS ONE's style requirements, including those for file naming.

**We have thoroughly checked and edited the manuscript based on the required style of PLOS ONE. Thank you for the comments.**

2. We suggest you thoroughly copyedit your manuscript for language usage, spelling, and grammar. If you do not know anyone who can help you do this, you may wish to consider employing a professional scientific editing service.
**We have thoroughly read the manuscript and grammatical and editorial errors have been corrected in “track changes” mode. Additionally, the language usage, spelling, and grammar have also been checked by native English language speaker (Professor Gary Wessel, Brown University, USA) whom we duly acknowledged in the Acknowledgments section of this manuscript. Thank you for the comments.**

3. Please ensure that you refer to Figure 2 in your text as, if accepted, production will need this reference to link the reader to the figure.

**The comment is accepted and the figure is now cited in the body of the revised manuscript.**

4. We note that Figure 1 in your submission contain map images which may be copyrighted. All PLOS content is published under the Creative Commons Attribution License (CC BY 4.0), which means that the manuscript, images, and Supporting Information files will be freely available online, and any third party is permitted to access, download, copy, distribute, and use these materials in any way, even commercially, with proper attribution. For these reasons, we cannot publish previously copyrighted maps or satellite images created using proprietary data, such as Google software (Google Maps, Street View, and Earth).

### Figure 1 was constructed using ArcGIS software. First, the shape file of the country and the regions were downloaded from the DVIA-GIS (https://www.diva-gis.org/gdata), which is a freely available computer program for mapping and for downloading and analyses of geographic data of any country. Then, the ArcGIS software was used to generate Figure 1. Hence, the Figure is not copyrighted.

**Response to Reviewer Comments**

**We thank the reviewer for their thoroughly reading the manuscript and for their thoughtful comments and constructive suggestions, which helped us to improve the quality of this manuscript. We have accommodated all the comments and accepted the suggestions. We have revised the manuscript using the track changes mode in MS Word.**

Reviewer #1: The manuscript by Enyew et al. seeks to address the complications associated with G x E interactions in sorghum, and reports of elite cultivars. The research has been rigorously performed. However, my few comments are as follows;

1. Sentences in the abstract should be carefully looked at, and must be concise as possible.

**The comment is accepted and the abstract is carefully revised.**

2. If possible, authors should recheck for grammatical errors. For instance line 53,.... be "used"; 65, ... it's grain yield "varies", and many more.

**We have carefully checked and revised the manuscript and corrected grammatical and editorial errors. The language usage, spelling, and grammar have also been checked and edited by native English language speaker (Professor Gary Wessel, from Brown University, USA). Thank you for the comments.**

3. Authors should ensure consistency of the tables and make sure they meet the requirements of the journal.

**We have checked the consistency of the tables and rearranged them according to the required style of PLOS ONE.**
